# A review of drivers of emerging infectious diseases within the wildlife-human-domestic animal interface in the West Pacific Tropics

**DOI:** 10.1016/j.onehlt.2026.101395

**Published:** 2026-04-02

**Authors:** Emilia A. Lastica-Ternura, Paul Horwood, Lee Francis Skerratt, Bruce Gummow

**Affiliations:** a Discipline of Veterinary Science, James Cook University, Townsville, QLD, Australia; b Department of Veterinary Clinical Sciences, College of Veterinary Medicine, University of the Philippines Los Banos, Laguna, Philippines; c One Health Research Group, Melbourne Veterinary School, Faculty of Science, University of Melbourne, Werribee, VIC, Australia; d Faculty of Veterinary Science, University of Pretoria, South Africa

**Keywords:** Emerging infectious diseases, Drivers, Socioeconomics and governance, Transboundary interfaces, West Pacific Tropics

## Abstract

The West Pacific Tropics (WPT) is a hotspot for disease emergence. Rich biodiversity, high species population density, susceptibility to climate change, rapid land use changes and high transboundary traffic, provide an ideal situation for disease spread, but their interaction in driving disease emergence is poorly understood. Studies related to drivers of emerging infectious diseases (EID) published between 2004 and 2024 were gathered through a document search using Web of Science, SCOPUS and MedLine. A PRISMA diagram was used to document the selection process, and track materials retrieved. A total of 23,235 articles were collated using EndNote™ (ver. 21.5) during the initial search. After removal of duplicates and grey literature, titles and abstracts of 16,442 articles were screened based on the exclusion and inclusion criteria, and 75 articles were sought for retrieval. Out of the 43 eligible reports for full paper review, 29 were excluded based on the exclusion criteria and 14 studies were included in the review. The small number of eligible studies was partially because documents chosen were limited to studies or topics within the WPT. Some territories were underrepresented which introduced some bias into the study. Included studies were analysed using a direct acyclic graph (DAG), which identified ten vectors that showed causal paths leading to EID. Socioeconomics and governance were key drivers of EID within wildlife-human-domestic animal transboundary interface in the WPT. By framing these interactions within a regional One Health systems perspective, the study shows that governance and socioeconomic factors act as cross-cutting determinants that influence multiple pathways of pathogen spillover and spread. Activities that promote increased proximity of wildlife to domestic animals and people serve to mediate disease transmission and spread. Harmonising wildlife trade regulation and strengthened territorial biosecurity measures through the adoption of regional best practices are recommended as key disease prevention strategies.

## 1 Introduction

The West Pacific Tropics (WPT) is a geographically and ecologically significant region defined primarily by its tropical climate, high biodiversity, and location on the western margin of the Pacific Ocean, generally encompassing the areas between the Equator and the Tropic of Capricorn. It is characterized by warm ocean currents, high annual rainfall, and the presence of numerous islands, including the large landmasses of New Guinea and the northern tip of Australia. It includes 23 countries, and territories, all of which possess a rich biodiversity, high species population densities, susceptibility to climate change, rapid land use changes and high transboundary traffic which, provide a unique environment for disease emergence and spread, making it of interest to epidemiologists [1].

Emerging infectious diseases (EID) impact health, food security, biodiversity and economic stability. In countries most vulnerable to the consequences of climate change and other stressors, these impacts could mean dire consequences to an already vulnerable population of animals and humans. Drivers of disease emergence need to be elucidated to understand and mitigate risk, particularly disease emergence at wildlife-human-domestic animal interfaces [1]. Rapid land use change and decreasing natural barriers between humans and wildlife is a major driver of the emergence of zoonotic pathogens and parasites [2], as in the case of vector-borne diseases [3], [4] and bat-mediated emerging diseases such as Nipah Virus [5].

Major drivers of diseases have been identified [6], [7] as a mixture of host, environment, pathogen and socio-political factors, with the latter being both a driver and magnifier of disease emergence and spread [6], [7], [8].

Previous studies of EID have identified broad ecological and anthropogenic drivers of zoonotic emergence and mapped global hotspots, but they provide limited insight into how these drivers interact within specific regional wildlife–human–domestic animal interfaces. This review advances the literature by synthesising evidence from the WPT and applying a directed acyclic graph (DAG) approach to identify causal pathways linking ecological, socioeconomic and governance drivers of disease emergence.

It aims to show that by framing these interactions within a regional One Health systems perspective, governance and socioeconomic factors act as cross-cutting determinants that influence multiple pathways of pathogen spillover and spread.

## 2 Methods

### 2.1 Evidence review

The review was performed by searching for studies related to drivers of emerging infectious diseases published between 2004 and 2024 gathered through a document search using Web of Science, SCOPUS and MedLine. The following prompts were used to come up with a more comprehensive search: “emerging communicable disease” OR “emerging infectious disease” OR “emerging infectious diseases” OR “re-emerging communicable disease” OR “re-emerging communicable diseases” OR “re-emerging infectious disease” OR “re-emerging infectious diseases” OR zoono* OR anthropozoon* OR “cross-species transmission” OR “interspecies transmission” OR “spill over” OR “spill back” OR “pathogen pollution”. Search duration was two months. Documents chosen were limited to studies or topics within the West Pacific Tropics, with countries and territories comprising the following: Philippines, Indonesia, Malaysia, Brunei and Singapore in Southeast Asia; Taiwan, Hongkong and Macau in East Asia; the islands of Papua New Guinea, Fiji, Solomon Islands, Vanuatu, New Caledonia, Tonga, Samoa, Kiribati, Tuvalu and Nauru in Oceania; the Pacific Islands of Mariana, Palau, Micronesia and Marshall Islands; and the Northern portions of Australia, comprised of Queensland and the Northern Territories, including Darwin. These territories were chosen because they share common traits, such as high biological diversity, susceptibility to climate change effects, and an increased susceptibility to emerging diseases [1].

Eligible articles passed through exclusion and inclusion criteria applied to the titles and abstracts of the papers (Supplementary Table 1). All duplicates were excluded, as well as papers that come from a single study, irrespective of the type of publication of these papers. Papers that describe causality and describe disease spread from wildlife to domestic animals, animals to humans and *vice versa* were considered eligible. Therefore, descriptions of new occurrences, strains or species, unless spread through interfaces, were excluded. Grey literature was not included in the review because they were not readily available in common academic search engines. The authors decided to exclude grey literature that was found because tracing the original document was difficult and many of them are not peer-reviewed. They do, however, form an important source of information in the WPT because of the limited number of peer reviewed publications from the region. This remains a weakness of this study.

**Fig. 1:**
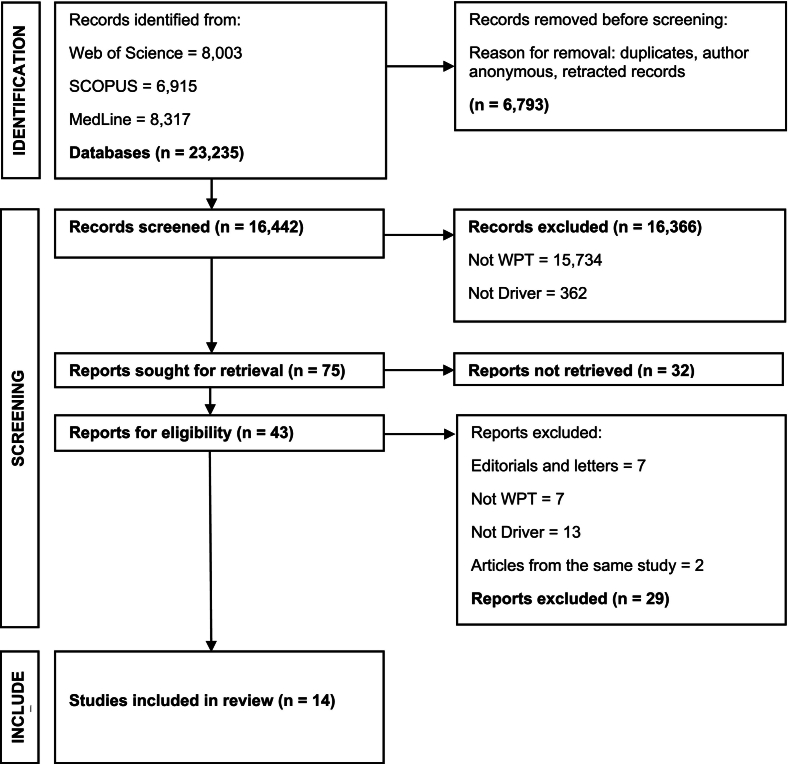
PRISMA diagram for identification, screening and inclusion of journal articles on drivers of EIDs in the West Pacific Tropics.

The PRISMA diagram was used to document the selection process, and track materials retrieved [9] (Fig. 1). The review is registered under the Open Science Framework (OSF) [10].

Key information such as author, title, date, and type of publication were recorded. The country or territory, species of concern, pathogens of interest and drivers identified were noted. All these information was tabulated in a spreadsheet for data recording and analysis.

## 3 Direct Acyclic Graph (DAG)

A direct acyclic graph (DAG) was generated for each of the articles included in the review. DAGs are a graphical method to understand relationships of exposures, outcomes, confounders, multipliers and other factors that may influence outcomes [11], [12]. Covariates related to the development and spread of diseases were identified and mapped on a DAG using the following assumptions: 1) drivers identified in each of the articles are treated as exposures; 2) diseases or pathogens were treated as the outcome, EID; 3) other covariates were plotted as descendants of either the exposures or the outcome. The direction of causal relationships was determined by how disease spread was described and explained within the included articles.

These individual DAGs were then consolidated to encompass all the articles in the review to arrive at a graph summarizing the drivers of EID within the wildlife-human-domestic animal interfaces in the WPT [13]. The generated graph was formatted to show the different covariates leading to EID, enabling a clearer illustration of how these covariates interact to bring about EID.

Using DAGitty.net, drivers (*n* = 7) “translocation”, “intensification”, “land use change”, “socioeconomics and governance”, “urbanization”, “wildlife trade” and “human-wildlife interaction” were linked to the single outcome “Emerging Infectious Diseases (EID)”. Ancestors of exposures (*n* = 10), or factors that influence these drivers, and ancestors of the outcome (*n* = 14), or factors that influence EID, were also identified.

Cyclic pathways were removed, and confounding bias was controlled by calculating one of the minimum adjustment sets (MAS) [11], which DAGitty.net automatically computes. After DAG construction, each vertex and arc were described (Table 1).

**Table 1:** Articles included in the review with the animals, diseases and EID drivers mentioned.

**Article**	**Animals**	**Diseases/** **Pathogens**	**Drivers**
Abdullah et al. (2024)	Pscittacines	Pscittacosis	Wildlife trade
	Turkey	SARS	Globalisation
	Humans	COVID	Translocation
			Regulation inconsistencies
Balasubramaniam et al. (2022)	Macaque	Influenza	Inter-species interactions
	Humans	Measles	
		Tuberculosis	
Blasdell et al. (2022)	Rats	Coronavirus	Urbanization
	Humans	Mammarenavirus	Habitat loss
		Orthohantavirus	
		Paramyxovirus	
		Bartonellosis	
		Leptospirosis	
		Toxoplasmosis	

Byrne et al. (2021)	Macaques	Malaria	Deforestation
	Vectors		Agriculture
	Humans		
Catlay et al. (2017)	Suidae	Avian paramyxovirus	Wild meat trade
	Cervidae	cercopithecine herpesvirus	Contact with wildlife
	Viviridae	Cowpox	Hunting
	Caprinae	Reston ebola	Butchering
	Pteropodidae	Hepatitis E	Consumption of wildlife
	Hystricidae	Highly pathogenic avian	Land use changes
	Ursidae	influenza	Human encroachment into wild habitats
	Cercopithecidae	Lymphocytic	Urbanization
	Felidae	choriomeningitis	
	Manidae	Nipah virus	
	Elephantidae	Orf	
	Squamata	Rabies	
	Testudines	Reovirus	
	Crocodylia	SARS coronavirus	
		Simian foamy virus	
	Galliformes	Simian type D retrovirus	
		Swine influenza virus	
		Anthrax	
		Bartonella	
		Brucella	
		Campylobacter	
		Chlamydophila	
		*Dermatophilus congolensis*	
		Edwarsiella tarda	
		*Erysipelothrix rhusiopathiae*	
		*E. coli*	
		*Francisella tularensis*	
		Leptospira	
		Mycobacterum tuberculosis	
		Pasteurella	
		Salmonella	
		Shigella	
		Streptococcus	
		Shigella	
		Streptococcus	
		Yersinia	
		Ancylostoma	
		Anisakidae	
		Balantidium	
		Cryptosporidium Enantomoeba	
		Giardia	
		Gnathostoma	
		Oesophagostomum	
		Pentastomidia	
		Sarcocystis	
		Spirometra	
		Stongyloides	
		Taenia	
		Toxoplasma gondii	
		Trichinella	
		Trichuris	
Diptyanusa et al. (2021)	Mosquito vector	Japanese encephalitis	Land use change
	Bats		
	Humans		
Fornace et al. (2016)	Pig-tailed macaques	Malaria	Deforestation Land use change
	Long-tailed macaques		
	Humans		
Hawkes et al. (2019)	Macaques	Malaria	Land use change
	Humans		
Leung (2024)	Monitor lizards	Alaria	Pet trade
	Snakes	Sparganosis	
	Frogs	Nematodes	
	Crocodiles	Armilifer	
	Iguanas	Ticks	
	Humans	Protozoa	
		Mites	
		Trypanosoma	
		Giardia	
		Cryptosporidium	
Mayfield et al. (2018)	Rodents	Leptospirosis	Poverty
	Pigs		Socio-demographic systems
	Humans		Exposure to livestock
Paez et al. (2017)	Giant flying foxes	Hendra virus	Climatic factors
	Humans		Population density
Paller et al. (2024)	Rats	Helminth parasites	Urbanization
	Humans		Land use change
			Broader distribution of intermediate hosts
Root et al. (2017)	Birds	Avian influenza	Increased human-animal interactions
	Humans		Socio-economic factors
Steiger et al. (2012)	Mosquito vector	Mosquito-borne diseases	Land use change
	Humans		Deforestation

## 4 Results

A total of 23,235 articles were collated using EndNote™ (ver. 21.5) during the initial search. After removal of duplicates, grey literature and articles with anonymous authors, 16,442 articles were screened based on the exclusion and inclusion criteria, and 75 articles were sought for retrieval. Thirty-two articles were not retrieved. Out of the 43 eligible reports for full paper review, 29 were excluded based on the exclusion criteria and 14 studies were included in the review, 11 of which were research articles, and three were reviews.

**Fig. 2:**
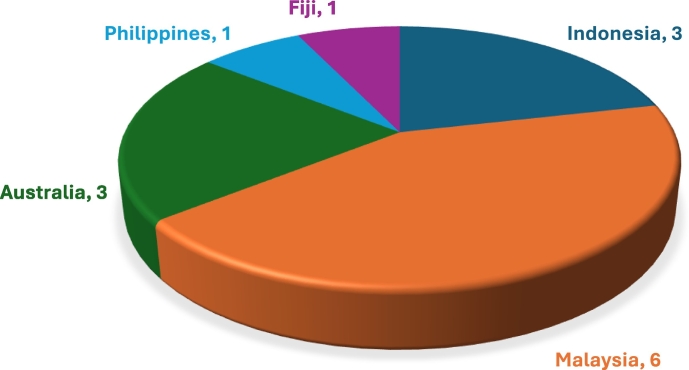
Out of 23 territories within the West Pacific Tropics, only five were represented, with Malaysia having the greatest number of articles (*N* = 14).

**Fig. 3:**
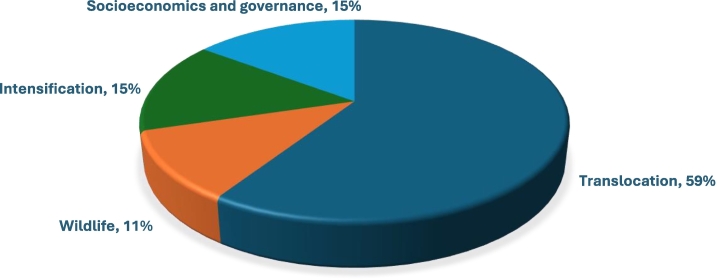
Drivers of EID in the transboundary interfaces within the WPT. Translocation was identified as a major disease driver, followed by socioeconomics and governance, intensification, and wildlife.

The 14 articles included in this review represented studies from five countries out of the 23 territories included in the Western Pacific Tropics, representing only 28% of all the territories of the region. Malaysia showed the greatest number of studies (*n* = 6), followed by Indonesia (*n* = 3) and Australia (n = 3), and the Philippines and Fiji, with one study each (Fig. 2). A total of 48 animals were discussed, with the majority focused on wildlife (73%, *n* = 35), followed by humans (25%, *n* = 12) and domestic animals (8%, *n* = 4) (Fig. 3). Most of the pathogens identified (*N* = 82) were viruses (34%, *n* = 28), followed by bacteria (28%, *n* = 23), parasitic worms (20%, *n* = 16) and protozoa (17%, *n* = 14). Two papers touched on ectoparasites (2%). Among the articles, EID drivers mentioned were translocation (59%, n = 14), intensification (16%, n = 4), socioeconomics and governance (16%, n = 4) and wildlife (12%, *n* = 3) (Fig. 3).

**Fig. 4:**
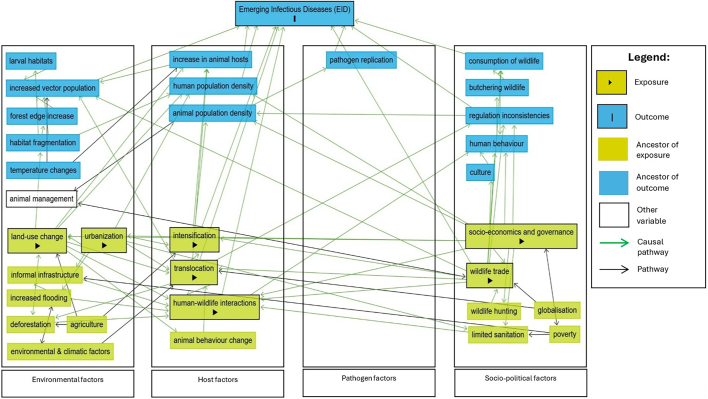
Direct Acyclic Graph (DAG) of drivers of EID within the wildlife-human-domestic animal interface in the WPT showing identified drivers (►) cause EID (I) through multipliers (blue) that increase likelihood of EID. Exposures were grouped according to environmental factors, host factors, pathogen factors and socio-political factors (Ndow et al., 2019). Of these, ‘socioeconomics and governance’ is shown to be both a driver and an amplifier of other known drivers. (For interpretation of the references to colour in this figure legend, the reader is referred to the web version of this article.)

Using DAGitty.net, exposures (*n* = 7) identified were: “translocation”, “intensification”, “land use change”, “socioeconomics and governance”, “urbanization”, “wildlife trade” and “human-wildlife interaction” as these were the drivers identified in the articles. Ancestors of these drivers (*n* = 10) were the following: for “translocation”: “environmental and climatic factors, ‘deforestation’, ‘animal behaviour change’ and ‘globalisation’; for ‘intensification’: ‘urbanization’ and ‘agriculture’; for ‘land use change’: ‘agriculture’, ‘deforestation’, socio-economics and governance”, “animal behaviour change” and “urbanization”; for “urbanization”: “socio-economics and governance”; for “wildlife trade”: “wildlife hunting”, “globalisation” and socio-economics and governance”; for “human-wildlife interaction”: “socio-economics and governance”, informal infrastructure”, “limited sanitation”, “increased flooding”, “deforestation”, “land use change”, “translocation” and “wildlife trade”. Many of these ancestors to the exposure shared direct pathways with other exposures (Fig. 4).

Mediators, or ancestors of the outcome (*n* = 14) between identified drivers and EID showed either direct causality or are themselves mediators as well. A total of 25 covariates and 310 causal paths were identified. Not all identified drivers in literature had direct causal paths to EID, such as, “land-use change”, “urbanization” and “socioeconomics and governance”, however, “socioeconomics and governance” was shown to have causal paths towards many mediators, exposures and ancestors of exposures.

“Poverty”, “agriculture”, “globalisation”, and “environmental and climatic factors” were not shown to have causal paths to EID or their drivers, which means that EID can occur with or without them. Similarly, “temperature changes”, while an ancestor of “EID”, does not have a causal path to it. All these factors were either ancestors, or descendants of identified exposures with causal paths to “EID”. This should be interpreted an inability to establish a causal pathway for these factors rather than evidence that no pathway exists, as the absence of a causal pathway in a model may reflect limitations in the available evidence.

Drivers were divided into environmental, host, pathogen and socio-political factors, and this was reflected in the DAG, which identified ten vectors that showed causal paths leading to “EID”, namely: “consumption of wildlife”, “human population density”, “human-wildlife interactions”, “increase in animal hosts”, “increased vector population”, “pathogen replication”, “regulation inconsistencies”, “wildlife trade”, “intensification” and “translocation”. Of these, “socioeconomics and governance” showed both direct or indirect causal paths to “consumption of wildlife, “human population density”, “human-wildlife interactions”, “regulation inconsistencies”, and “wildlife trade”. Half of all the drivers identified by the DAG had causal paths to EID.

## 5 Discussion

The five countries represented in this review accounts for 28% of all territories of the WPT. This illustrates an imbalance of research in the region. Although studies were made in areas such as the Pacific Islands, many of these are grey literature, consisting of workshop reports, and research that did not make it to widely distributed high-impact journals. Because some territories were underrepresented, EID drivers from these areas may not be properly identified. This may affect conclusions regarding dominant drivers identified in the review, such as ecological factors, and socioeconomics and governance. Funding sources of the articles included in the eligible papers showed external research support for many of these projects, which were mostly funded by the United States of America or the United Kingdom. A disparity in funding resources both for research and dissemination exists for territories within the WPT, where funding is available only for research on diseases involving wealthier countries [14], or those that have researchers from those countries. There is a need for a larger area of the West Pacific to be studied, or for completed studies to make it into widely circulated journals, so that information regarding these territories can be included in informed international policy creation.

Thirteen papers tackled wildlife either as intermediate or definitive hosts of EIDs [15], [16], [17], [18], [19], [20], [21], [22], [23], [24], [25], [26], [27], indicating awareness on the possible role of wildlife in disease spread, however only 11% considered that wildlife drives EID spread [17], [23], [24]. Limited knowledge on wildlife and their pathogens indicates high biodiversity as a driver for disease emergence [28]. High species diversity in proximity can increase pathogen exchange and lead to genetic mutations [29], especially if they interact closely with each other.

Viruses were 34% of the pathogens mentioned [16], [17], [18], [19], [20], [21], [22], [23], [24], [25], showing that studies in the WPT explore viral diseases more than studies of other pathogens.

### 5.1 Translocation, land-use change and urbanization

Translocation was identified by all the articles as a major disease driver [4], [15], [16], [17], [18], [19], [20], [21], [22], [23], [24], [25], [26], [27]. It is the movement of people, animals and animal products from one location to another [6], [7], [30], [31], [32], bringing with them pathogens that may lead to pollution or spill-over. This may be in the form of animal and human migration and tourism, and pet, meat and by-product trade [17], [26], [30], [33]. The increasing popularity of exotic pets can trigger the spread of unknown pathogens, as many of these are harboured by less studied animals, such as reptiles, amphibians or invertebrates [26], [33]. Most work on zoonoses were done on mammals and overlooked the importance of reptiles as threats to human health. This is because of the greater affinity of humans to other mammals, such as most food animals and pets [26]. Wildlife releases, whether intentional through reintroduction programs for conservation, or exotic pet releases are also another form of translocation that may cause pathogen pollution into wild spaces [30], [33], [34]. Apart from EID, translocation also had direct causal paths to human-wildlife interactions, increase in animal hosts, increased vector population, regulation inconsistencies, deforestation, and intensification [17], [21], [25], [35].

Land use change, in the form of deforestation, agricultural expansion or urbanization have been proposed as a main driver of disease emergence, particularly of vector-borne [15], [21], [22], [35], or rodent-borne diseases [24], [27] as it has causal paths to animal behaviour change, habitat fragmentation, human population density, human-wildlife interactions, increase in animal hosts, deforestation and translocation [4], [15], [17], [20], [21], [22], [27], [35]. Although an environmental factor of disease emergence [7], land-use change is closely related to human movement and result in human and animal translocations as vectors and wildlife are forced to adapt to new environments, carrying pathogens with them, and forcing different species in proximity [15], [21], [22], [27], [35]. Whilst farms may prevent disease spread through strict and effective biosecurity, wildlife are free-roaming and may easily cross into human habitations through translocation in the form of migration, wildlife trade, or consumption of wild animal products [6], [7], [30], [31], [32].

Urbanization is creeping human colonization of intermediate and wild spaces [24], [27] and is a collective result of habitat or land use change, host displacement, increased host density, altered intra or inter specific interactions and behaviour/movement patterns that induces population booms of rodent-borne pathogens. This, coupled with poverty and over stretched social systems, is a challenge to many countries in the WPT, particularly those without preventative health and biosecurity systems fully in place. Urbanization has direct causal paths to human population density, increased flooding, informal infrastructure, land-use change, limited sanitation, intensification and translocation [17], [24], [27]. Thus, rapid urbanization in the region can have dire implications for disease emergence, especially for diseases brought about by insect vectors, rodents or pets [7], [15], [24], [27].

Environmental changes such as climate change, drought, or flooding can influence behaviour changes in animals and trigger translocation [21], and any event that brings about massive translocation of animals and people may lead to disease emergence, as translocation leads to increased wildlife-human-domestic animal interaction [23], [25], [26], [35].

The WPT includes some of the countries that are most susceptible to climate change, and where expansive land use changes and rapid urbanization occur [1], [36]. Countries within the WPT may not only need to strengthen biosecurity locally, and within their respective territories, but also work for a more effective implementation at the regional level to halt EID spread.

### 5.2 Intensification in wildlife, domestic animal and human spaces

Causal paths that lead to intensification are wildlife trade, agriculture, translocation and urbanization [4], [15], [17], [23], [24], [26], [27], [35]. Intensification pushes organisms in ever smaller spaces and is more commonly attributed to food animal systems, where livestock and poultry production are maximised, resulting in higher population densities and promoting pathogen exchange between conspecifics [6], [17], [18], [21], [23]. Pathogen replication was shown to have a direct causal path to EID, and this was related to increased populations of both vectors and hosts [4], [15], [35]. Intensification has direct causal paths to EID, animal population density and increase in animal hosts, and pathogen exchange in this context will also promote genetic mutation, resulting in robust and resistant pathogens that can infect a wider host range [37], which may spill over adjacent farms and wildlife through trade or migration [38], [39], [40], [41].

Like farm animal intensification in commercial farms, which increases interspecific pathogen exchange and genetic mutations, wildlife intensification in ecological islands [42] and highly fragmented habitats may lead to an increased incidence of interspecies disease spread [29], [41]. However, in contrast to farming systems which are contained, wildlife and their pathogens can spill and promote pathogen transport to new areas, including human habitats [4], [18], [41].

For humans, rapid urbanization can cause population concentrations in limited city spaces, forcing them in proximity with intermediate hosts such as pets, rodent pests and stray animals [24], [26], [27], [43]. In territories with inadequate instruments for social services, biosecurity and disease outbreak response, negative consequences of rapid urbanization related to health and safety are often not adequately prioritized [1], [8], [36], [43], [44]. Biosecurity and disease prevention in densely populated environments such as farms, cities and highly fragmented wild habitats are essential for preventing spillover beyond these settings.

### 5.3 Socioeconomics and governance as a magnifier of EID

Socioeconomics and governance were identified by eligible literature as “political systems” that drive EID [16], [17], [19], [25]. Socio-political drivers figure significantly in disease emergence because much of the decision-making, policy, and budgeting surrounding land use change, urbanization, and any form of economic development, including those concerning trade, biosecurity and health are often based on socioeconomic factors [1], [7], [8], [19]. Poverty was seen as a driver of disease spread, as it paves way for a snowballing of many other challenges to disease prevention, control and treatment [19], [24]. Poverty on its own did not show causal pathways to EID. On the other hand, socioeconomics and governance directly influence human population density, human-wildlife interactions, regulation inconsistencies and wildlife trade [7], [16], [17], [19], [25], which all show direct causal paths to EID. Apart from these, socioeconomics and governance show causal paths to animal population density, human behaviour, land-use change, limited sanitation, wildlife hunting, culture, urbanization and poverty, which also shows a direct causal path back to socioeconomics and governance [16], [17], [19], [25], [43].

Socioeconomics and governance also influence how countries respond to challenges in biodiversity conservation, of which the WPT has many [36], [45]. Because many countries in the WPT are sources and conduits of wildlife trade, strong political will is needed to address gaps in systems that promote regulation inconsistencies surrounding trade in wildlife and lapses in biosecurity [19], [24], [25], [46].

Industries that result in habitat degradation are often backed by governments that earn from these same industries [47]. Countries with poor political systems do not have the instruments in place to protect biodiversity and the ill effects of destructive industries, causing an exacerbation of these outcomes, including the inability to efficiently respond to epidemics [1], [7]. Socioeconomics and governance can be both a driver and a multiplier of EID, as poor political systems can trigger other drivers to occur, resulting in an avalanche of other drivers and intensifying disease occurrence and spread.

### 5.4 Wildlife as a driver for unknown pathogen spread

Wildlife is often implicated as a source of emerging infections, however, only three papers in the review implicate wildlife as a driver for EID [17], [23], [24]. Despite the many studies about the role of wildlife in disease spread, there is still much to learn, considering the many species that have yet to be discovered and studied, because an estimated 70% of pathogens are predicted to come from wildlife [33], [43]. Vectors with causal paths to wildlife trade are animal management, socioeconomics and governance, and globalisation [17], [25], [26].

Wildlife trade is seen as an emerging cause of disease spread and biodiversity loss [17], [25], [26], [32], [33], [48], as it pushes intensification, translocation, and is driven by socio-economic influences [17], [25], [26], [30], [32], [33]. Much emphasis is on illegal wildlife trade, especially when animals that cross borders do not undergo the usual biosecurity measures that legal trade imposes [17], [49]. Confiscated wildlife that end up in rescue centres have been found to have been afflicted with a host of diseases, some of which are zoonotic [33], [48], [50], [53]. Disease risk related to wildlife trade also includes how wildlife markets and captive wildlife facilities are managed [17], [25], [50], [52], [53]. Regulation inconsistencies among countries involved in the wildlife trade networks promote an increase in EID, and countries within the WPT are known origins and conduits of wild animal trade [17], [25], [54]. Whilst wildlife *per se* do not cause EID, factors that promote human-wildlife interactions may serve to mediate disease transmission and spread [15], [17], [23], [24], [25], [34]. Wildlife conservation practices such as releases and reintroductions may pose a threat to wildlife in natural habitats, particularly if protocols for release are not strictly followed [30], [33], [49], [50], [51], [52], [53].

Threats to biodiversity are the same components that drive the emergence of infectious disease and thus environmental crises lead to health crises [25], [29], [54]. Increased land use changes that lead to habitat loss can eventually result to threats to species conservation, initiating a knock-on effect that includes disease emergence and spread [15], [45]. Conversely, species conservation can provide numerous benefits as a preventive for disease emergence, an insurance for food security and economic stability [45], [54], [55].

## 6 Conclusion

These findings highlight the central role of socio-economic and governance systems in shaping emerging infectious disease risk at the wildlife–human–domestic animal interface in the WPT. Strengthening One Health readiness therefore requires coordinated policy action that harmonises wildlife trade regulations and enforcement across countries, building on existing frameworks such as the Convention on International Trade in Endangered Species of Wild Fauna and Flora, while strengthening territorial biosecurity measures at borders and wildlife markets. In parallel, governments should prioritise integrated surveillance at high-risk interfaces, linking wildlife, livestock and human health systems through collaboration with organisations such as the World Health Organization and the Food and Agriculture Organization, to enable earlier detection of spillover risks. The development of measurable governance indicators aligned with initiatives such as the Global Health Security Agenda would allow countries to monitor cross-sector coordination, regulatory compliance and biosecurity capacity, thereby strengthening regional preparedness for emerging infectious diseases.

### 6.1 Research gaps

The following research gaps were identified and ranked based on feasibility and urgency: 1) increase visibility of scientific output from less represented territories; and 2) increased research efforts on the impact of invasive species in disease transmission in relation to wildlife trade.

Only 28% of all territories of the WPT formed part of the review, showing a gap in reporting, which could cause regional and global decision-makers to make biased decisions about the region's vulnerability to emergence of diseases and how to mitigate this [14]. There is a lack of information in the WPT on how diseases spread within the region. Most of the papers retrieved were reports on new species, strains or new host species [52], [53], [58], [59]. While these are important, factors that drive these diseases to spread are equally important. Grey literature from government-related projects may reach a larger audience if these were made available on a wider scale and are accessible to researchers and decision-makers. Further information is needed from underrepresented territories of the WPT, particularly those which are most vulnerable to climate change and disease threats. This may be possible if grey literature is published in peer-reviewed journals or presented at scientific venues.

Apart from those that mentioned stray animals in urban areas [24], [27], the impact of invasive species was not discussed in any of the articles eligible for review. Invasive animals were identified as one of the things that link biodiversity and human health [29]. It should be noted that rodent vectors are mostly invasives, and other invasive species may not only carry diseases [19], [27], but also cause ecological imbalance through predation of endemics or resource competition with native species [56], [57]. The contribution of wildlife trade in the introduction of invasive species should be investigated, as inadvertent releases could pose serious biosecurity and conservation threats in the future. Research exploring the role of invasive species in disease transmission, particularly in relation to wildlife trade should also be pursued, as wildlife trade is very active in the region.

Biosecurity within the territorial units could help stem influx of invasive species but can also be an effective way to prevent diseases. This involves collectively working for biosecurity in the region, beyond the efforts to strengthen it within each of their territorial units [48].

The One Health approach could only work if local points of view and cultural perspectives are seen to play a role in understanding systemic influences of disease spread. Thus, first-hand accounts from these territories are essential for a wider understanding of how these systems operate. This will require regional understanding and cooperation, and activities that promote greater awareness of the drivers of EID in the WPT, so that One Health can be promoted throughout the region.

## CRediT authorship contribution statement

**Emilia A. Lastica-Ternura:** Writing – review & editing, Writing – original draft, Software, Methodology, Formal analysis, Data curation, Conceptualization. **Paul Horwood:** Writing – review & editing, Validation. **Lee Francis Skerratt:** Writing – review & editing, Validation, Conceptualization. **Bruce Gummow:** Writing – review & editing, Validation, Supervision, Methodology, Conceptualization.

## Funding statement

This work was supported by funding from the Philippine's Department of Science and Technology (DOST) though their Science Education Institution Grant, and the 10.13039/501100014166Philippine Council for Agriculture, Aquatic and Natural Resources Research and Development (PCAARRD) in cooperation with the 10.13039/501100000974Australian Centre for International Agricultural Research (ACIAR).

## Declaration of competing interest

The authors declare no conflicts of interest.

## Data Availability

Data will be made available on request.
