# Supplementary material for: A review of drivers of emerging infectious diseases within the wildlife-human-domestic animal interface in the West Pacific Tropics
Source: One Health. 2026 Apr 2;22:101395. doi: 10.1016/j.onehlt.2026.101395 (PMC13090146; doi:10.1016/j.onehlt.2026.101395)
Supplement: Supplementary file 1 — Supplementary material [file mmc1.docx]

Table 1. A summary of inclusion and exclusion criteria used to select drivers of emerging infectious diseases in the WPT

| **Criteria** | **Description** |
| --- | --- |
| Inclusion | Papers from and about territories in the West Pacific Tropics, such as Philippines, Indonesia, Malaysia, Brunei and Singapore in Southeast Asia; Taiwan, Hongkong and Macau in East Asia; the islands of Papua New Guinea, Fiji, Solomon Islands, Vanuatu, New Caledonia, Tonga, Samoa, Kiribati, Tuvalu and Nauru in Oceania; the Pacific Islands of Mariana, Palau, Micronesia and Marshall Islands; and the Northern portions of Australia, comprised of Queensland and the Northern Territories, including Darwin  Papers about diseases described as emerging, infectious, zoonoses, anthropozoonoses  Papers describing diseases transmitted across species, are spill overs, spill backs or are pathogen pollution  Papers describing disease drivers, causality and spread, such as translocation, intensification, land use change, socioeconomics and governance, urbanization, poverty, wildlife trade or human-wildlife interaction |
| Exclusion | Papers describing diseases in areas outside the West Pacific Tropics  Papers describing new species and strains without disease transmission descriptions  Duplicate articles, including those that belong to the same study  Grey literature, such as government or development project reports |
